# OptoGap is an optogenetics-enabled assay for quantification of cell–cell coupling in multicellular cardiac tissue

**DOI:** 10.1038/s41598-021-88573-1

**Published:** 2021-04-29

**Authors:** Patrick M. Boyle, Jinzhu Yu, Aleksandra Klimas, John C. Williams, Natalia A. Trayanova, Emilia Entcheva

**Affiliations:** 1 Department of Biomedical Engineering, Johns Hopkins University, Baltimore, MD USA; 2 Institute for Computational Medicine, Johns Hopkins University, Baltimore, MD USA; 3 Department of Bioengineering, University of Washington, Seattle, WA USA; 4 Institute for Stem Cell and Regenerative Medicine, University of Washington, Seattle, WA USA; 5 Center for Cardiovascular Biology, University of Washington, Seattle, WA USA; 6 Alliance for Cardiovascular Diagnostic and Treatment Innovation, Johns Hopkins University, Baltimore, MD USA; 7 Department of Biomedical Engineering, Stony Brook University, Stony Brook, NY USA; 8 Department of Biomedical Engineering, George Washington University, 800 22nd Street NW, Suite 5000, Washington, DC 20052 USA

**Keywords:** Computational biophysics, Biomedical engineering, Biophotonics, Optogenetics, High-throughput screening, Fluorescence imaging

## Abstract

Intercellular electrical coupling is an essential means of communication between cells. It is important to obtain quantitative knowledge of such coupling between cardiomyocytes and non-excitable cells when, for example, pathological electrical coupling between myofibroblasts and cardiomyocytes yields increased arrhythmia risk or during the integration of donor (e.g., cardiac progenitor) cells with native cardiomyocytes in cell-therapy approaches. Currently, there is no direct method for assessing heterocellular coupling within multicellular tissue. Here we demonstrate experimentally and computationally a new contactless assay for electrical coupling, OptoGap, based on selective illumination of inexcitable cells that express optogenetic actuators and optical sensing of the response of coupled excitable cells (e.g., cardiomyocytes) that are light-insensitive. Cell–cell coupling is quantified by the energy required to elicit an action potential via junctional current from the light-stimulated cell(s). The proposed technique is experimentally validated against the standard indirect approach, GapFRAP, using light-sensitive cardiac fibroblasts and non-transformed cardiomyocytes in a two-dimensional setting. Its potential applicability to the complex three-dimensional setting of the native heart is corroborated by computational modelling and proper calibration. Lastly, the sensitivity of OptoGap to intrinsic cell-scale excitability is robustly characterized via computational analysis.

## Introduction

Intercellular coupling is a fundamental form of communication between cells, essential for the synchronization of physiological processes in different organs. Pathologically altered coupling or the emergence of de novo coupling between host and donor cells are problems of interest in many cardiac applications, e.g., during cell delivery and cell integration for cardiac repair therapy^1,2^. Specifically, interactions between cardiomyocytes and fibroblasts are of interest, especially the pro-arrhythmic increase in coupling as the latter transition to myofibroblasts^3–6^. Furthermore, in cell therapy, it is critical to be able to understand the level and time course of coupling of donor stem-cell-derived myocytes to host cardiomyocytes^7,8^.

Electrical coupling in cardiac tissue is mediated primarily by low-resistance paths formed by gap-junctional proteins (connexins), that can link cardiomyocytes (CMs) to each other and to non-cardiomyocytes (nCMs), such as fibroblasts. Qualitative and quantitative methods, e.g., immunofluorescence, messenger RNA quantification and Western blots, are often used to assay connexin expression levels as a surrogate measure of coupling, but they do not provide functional information. A method for direct quantification of cell–cell coupling within the multicellular tissue context is highly desirable.

## Existing methods for assessment of intercellular coupling

**Figure 1:**
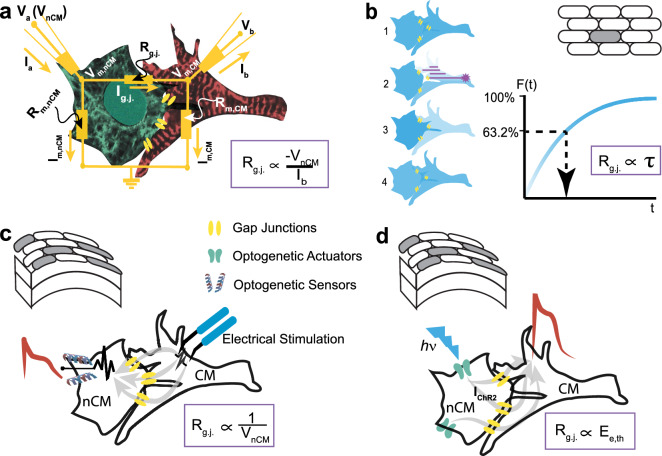
Existing and proposed methods for assessing electrical intercellular coupling. (**a**) Dual cell patch clamp measures the gap junction current based on equivalent circuits between two connected cells, a cardiomyocyte, CM (red) and a non-cardiomyocyte, nCM (green). After establishing an equilibrium (V_a_ = V_b_ = V_m,CM_ = V_m,nCM_) to eliminate junctional current, a voltage step is applied to one of the cell (e.g. V_nCM_) prompting a compensatory junctional current that can be recorded. Specifically, V_a_ acts as a stepping voltage source, the differential voltage (between V_a_ and V_m,nCM_) causes current I_a_ to be injected into the two-cell circuit that splits into I_m,nCM_ (nCM hence experiences depolarization) and I_g.j_, which flows out as –I_b_ and can be measured. The gap junctional resistance is proportional to the imposed voltage clamp, divided by the measured compensatory current. (**b**) Using low-molecular weight fluorescent dyes (1), GapFRAP infers coupling from the recovery of fluoresce in a target cell after it is subjected to photobleaching (2,3); the gap-junctional resistance is directly proportional to the time constant of recovery due to dye diffusion from neighbouring cells (4). The method is applicable to 2D multicellular settings. (**c**,**d**) Optogenetic methods offer new ways for assessing heterocellular coupling in the native tissue setting. (**c**) In the “optogenetic-sensor” variant, coupling is typically confirmed by measuring membrane potential fluctuations in nCMs (V_nCM_), expressing GEVI/GECI indicator and connected to CMs undergoing excitation (see Suppl. Figure 1 for quantitative details). (**d**) In the “optogenetic-actuator” approach presented here, coupling can be quantified by the light needed to trigger excitation in the CMs via the light-sensitive nCMs, i.e. E_e, th_.

Currently, no direct method exists for quantification of coupling in multicellular tissue. The “gold standard” for coupling measurements is the dual-cell patch clamp (Fig. 1a). It measures the gap junctional current between two connected cells, such as a CM and an nCM. Using a simplified equivalent circuit for the cell pair, one can quantify the equivalent gap junction conductance (1/R_g.j._)^9^. This method is strictly limited to isolated cell pairs with relatively high coupling resistance^10,11^; it is not applicable to the native multicellular setting and certainly not scalable.

For multicellular preparations, a class of indirect methods has been developed, which track the passive spread of low-molecular-weight dyes. The premise is that the diffusion of a small (gap-junction-permeable) molecule can be used as an indicator of the transmission of electrical signals between cells under certain assumptions. Several techniques fall into this category, notably fluorescence recovery after photobleaching (gapFRAP) (Fig. 1b)^11,12^, dye-injection, scrape loading and “local activation of molecular fluorescent probe" (LAMP)^13^. The measured variable is either the recovery of a fluorescent molecule in a photo-bleached cell (area) or the diffusion of an injected dye or activated uncaged fluorescent tracers from or to neighbouring cells. The time constant (τ) of recovery or spread correlates with junctional permeability^11^. Limitations include the interpretation of time constants, which is neither standardized nor absolute; the extraction of τ, which depends on the mathematical model used^14,15^; and the use of dyes of different molecular weight and diffusion rate, which can lead to different time constants for the same model and the results of which need to be calibrated. The benefit of these methods is their applicability to a multicellular setting, unlike the dual-cell patch clamp. GapFRAP in particular is a convenient approach, yet the derived τ lacks a direct relationship to the electrical conductance and dye permeability through gap junctions is not a definitive indicator of electrical coupling^16^. Furthermore, this class of methods is not directly extendable to three-dimensional tissues and not easily scalable, i.e. typically, a single cell/site is being manipulated at a time.

## Scalability and coupling metrics using optogenetic methods

**Figure 2:**
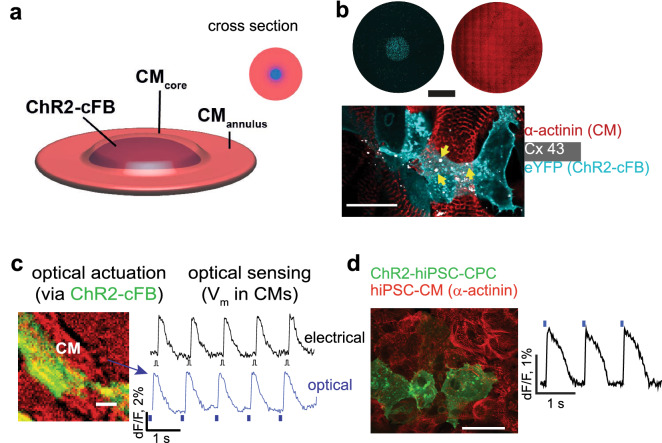
Experimental model of coupling optogenetically-modified nCMs and non-modified CMs. (**a**) Schematic view of the two-layer patterned co-culture of ChR2-cFB and CMs used in this study. A patterned smaller-diameter region of ChR2-cFB (blue core) forms the first layer, covered by a larger CM layer (red circle). (**b**) Immunofluorescent images of ChR2-cFB reported by eYFP (cyan) and CM labeled by α-actinin (red). Black scale bar is 4 mm. Yellow arrows indicate gap junctions (Cx43, white) detected between the two layers of CM and ChR2-cFB. White scale bar is 50 µm. (**c**,**d**) Functional confirmation of electrical coupling between light-sensitized nCMs and non-transformed CMs by optical voltage sensing. (**c**) Optical recording of electrically (5 ms) or optically-triggered (10 ms pulses, 0.31 mW/mm^2^ 470 nm light) action potentials in non-transformed CMs, co-cultured with ChR2-cFB (eYFP) from neonatal rat hearts. Scale bar is 50 µm. (**d**) Similarly, functional confirmation of electrical coupling is illustrated between light-sensitized human cardiac progenitor cells (ChR2-iPS-CPC) and non-transformed human iPSC-CMs (light pulses were 20 ms, 0.03 mW/mm^2^ 470 nm). Scale bar is 50 µm. This experiment was done in a 96-well format using all-optical electrophysiology.

Optogenetic tools (genetically-encoded light sensitive actuators or sensors) offer scalability, as well as cell specificity and can be used to characterize heterocellular electrical coupling, such as between CM and nCM. There are two options: (1) in an “optogenetic sensor” variant, nCM-specific expression of optogenetic sensors of voltage or calcium can be used to uncover heterocellular coupling (Fig. 1c), or (2) in an “optogenetic actuator” variant, nCM-specific expression of an optogenetic voltage actuator can be used (Fig. 1d). While the advantages of optical methods, such as high resolution, parallelism and scalability, are well-documented, no prior study has examined if these new optogenetics-inspired methods can be quantitative in assessing cell–cell coupling. A 2019 study by Wu et al.^17^, which appeared after a preprint of this work was deposited on bioRxiv in 2017^18^, is optogenetics-inspired and similar in concept to our approach. It used ArchT, an inhibitory opsin, and long light pulses, while optically measuring the response (calcium) in neighbouring cells to infer cell–cell coupling, similar to other sensor-based methods, with certain limitations, as described below.

Genetically-encoded calcium indicators (GECI), capable of reporting the activity of selected cells, surrounded by host cells (under external pacing or during intrinsic activities) have been applied to study the electrical integration of grafted pluripotent stem-cell-derived cardiomyocytes in animal models^8,19,20^. In these studies, coupling was assessed in a binary way (presence or absence) by the similarity in the frequency response between the host and donor cells, reported by optogenetic and conventional indicators. This approach may not be very specific because synchrony of responses is possible in the absence of electrical coupling, as exemplified for substrate-mediated mechanical coupling of distant cardiomyocytes, for example^21^. There is interest in employing genetically-encoded voltage indicators (GEVIs) to report in a more direct way heterocellular coupling in the intact heart, albeit still qualitatively^22^. The imperfect promoters currently used to target non-myocytes complicate the interpretation of results on coupling in vivo. We simulate the scenario of coupling such GEVI-expressing nCMs (cardiac fibroblasts used as an example) and CMs using the MacCannell model^23^ (Fig. 1c and Suppl. Figure 1) and assuming an ideal GEVI. The measures (of the voltage response of the two cell types) that are most useful as quantitative reporters of electrical coupling are shown. The inset of Suppl. Figure 1b indicates that the amplitude of the voltage reported by the GEVI in the nCM can quickly pass the threshold for detection (> 20 mV) at gap junctional values < 1 nS. Indeed, if nCM action potentials are used as indication of nCM–CM coupling, such coupling may be inferred even for very low values, below the ones normally considered meaningful^24,25^. This is an important limitation of the optogenetic-sensor method, using either GEVI or GECI, as in^17^. The modulation (shortening) of the action potential duration in either the nCM or the CM can be used as another surrogate measure of coupling with better sensitivity (extended to higher gap junctional values), as shown in Suppl. Figure 1c,d. It is important to note that these effects depend on the resting membrane potential of the cells and calibration may be challenging; a further practical difficulty to extract information from the GEVI-reported voltage traces is the still-limited signal-to-noise ratio of most of these indicators.

We hypothesized that a method using optogenetic actuators instead (Fig. 1d) may provide distinct advantages compared to the existing techniques. When combined with an optical readout to achieve an all-optical interrogation^26^, the method is highly parallel, i.e., can report coupling over different regions and many samples simultaneously. In contrast to the study by Wu et al.^17^, we focus on the actuator in the all-optical interrogation and show that the light power (E_e,th_) needed to stimulate (light-insensitive) excitable cells, e.g., CMs, through an opsin-expressing nCMs can be used as a quantitative measure of heterocellular coupling. This optogenetics-based coupling assay, which we term OptoGap, is applicable to a variety of coupled cell types, including human iPS-derived progenitor cells and human iPS-cardiomyocytes (Fig. 2), but for the rest of this report, primary rat cFB and CMs are used as an example experimental model. This work has been presented in abstract form^27^ and deposited on biorxiv^18^.

## An in vitro coupling model of ChR2-cFB and CM

To demonstrate OptoGap, an in vitro multicellular system was designed, consisting of a patterned region of cardiac fibroblasts expressing the light-sensitive actuator, channelrhodopsin-2 (ChR2-cFB), and a second layer of ventricular CM on top (Fig. 2a,b). Optimized generation of light-sensitive fibroblasts, ChR2-cFB, yielded a consistent expression efficiency of > 50%, Suppl. Figure 2^28^. In this model, cFB and CM tend to make gap junctions in the axial direction (not in-plane). Panoramic image of the immunolabeled samples confirmed the cell pattern and Cx43’s presence in the core between ChR2-cFB and CM (Fig. 2b). The conduction properties in this macroscopic experimental model are characterized in detail in the Suppl. Figure 3).

Consistent with our “tandem-cell-unit” method of optogenetic stimulation^25,29^, we confirmed that the ChR2-cFB can trigger global activation in the non-transformed CMs (Fig. 2c). Evidence for such in vitro coupling between CM and cFB has been provided by multiple studies^5,6,30^. To illustrate the more general applicability of the method, we also examined other cell types, including coupling between human iPS-derived cardiac progenitor cells, which were made light-sensitive, and iPSC-CMs. Optical stimulation of the iPSC-CMs via the ChR2-expressing cardiac progenitor cells was documented in this heterocellular model that has relevance to cell therapy (Fig. 2d).

## OptoGap: implementation and validation against gapFRAP

**Figure 3:**
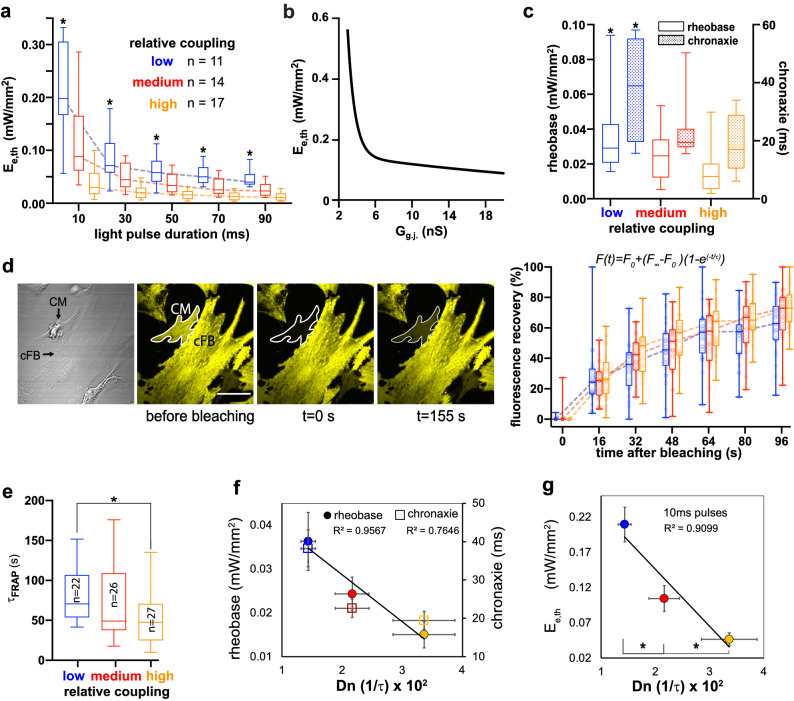
OptoGap performance and validation. (**a**) OptoGap response is quantified in terms of excitation threshold irradiances, E_e,th_, which vary as a function of optical pulse duration for the patterned experimental model from Fig. 2a,b (ChR2-cFB and CM), shown at three levels of gap junctional coupling strength. * indicates significant differences (p < 0.05) detected between the different coupling strengths: low (n = 11) medium (n = 13) and high (n = 16); normality of these distributions was confirmed. (**b**) A computational model of coupling between CM and ChR2-cFB predicts a distinct relationship between E_e,th_ and gap junctional coupling G_g.j._. Extended simulation results are shown in Supp. Figure 4. (**c**) OptoGap confirms computational predictions—illustration using key parameters: rheobase and chronaxie, extracted from the strength-duration curves in (**a**) by fitting the relationship $\frac{rheobase}{{1 - e^{{\frac{ - xlog(2)}{{chronaxie}}}} }}$. * indicates significant difference between the low coupling strength and the other two coupling strength. (**d**) gapFRAP evaluation of coupling between ChR2-cFBs and CMs and recovery curves for these for the three levels of coupling. Scale bar is 50 µm. Dye fluorescence intensities obtained from sequential images of samples at three controlled coupling levels. In FRAP curves on the right are constructed using n = 22 samples for low, n = 26 samples for medium, and n = 27 samples for high coupling; the formula used to extract time constant τ_FRAP_ is shown. (**e**) Extracted τ_FRAP_ for each coupling condition is plotted, * significant difference, p < 0.05. (**f**) Cross-validation of OptoGap using standard gapFRAP. The lines are linear regression fitting rheobase and chronaxie mean value points, respectively. Data are presented as mean ± SE; the experimental data distributions for the rheobase and the chronaxie are shown in (**c**), while the data distribution for Dn is shown in Suppl. Figure 6. (**g**) OptoGap yields the highest sensitivity to coupling (higher than gapFRAP) at short pulses; E_e,th_ for 10 ms pulses detected significant differences between all three coupling conditions (*); data presented as mean ± SE. Data distributions in panels (**a**), (**c**), (**d**) and (**e**) are presented as box-whisker plots including the median, the lower and upper quartile range, with whiskers covering the min–max range of the data.

After confirming the functionality of the experimental model to study heterocellular coupling, we created a range of coupling conditions. In addition to default (control) coupling, “low” or “high” coupling conditions were produced by using uncoupling agent (0.5 mM heptanol) or a coupling-boosting agent (1 mM sodium 4-phenylbutyrate), as we have previously reported^31^. Demonstration of OptoGap was done at the macroscale, by applying global illumination with blue light (470 nm) at the core area and confirming a wave of excitation originating from the core and propagating radially; pacing at 1 Hz with variable pulse duration had to yield full capture of at least ten consecutive beats in order to determine E_e,th_. Upon optical stimulation, for the three coupling conditions, we were able to detect three distinct strength-duration curves (Fig. 3a), supporting the model-informed idea (Fig. 3b) that the light power used to excite, E_e,th_, can serve as a metric of coupling strength between the cFB and CMs. In increasing order of pulse duration, E_e,th_ of the low-coupling group ranged from 0.045 to 0.21 mW/mm^2^; that of the control group ranged from 0.024 to 0.105 mW/mm^2^; and that of the high coupling group ranged from 0.016 to 0.047 mW/mm^2^. Suppl. Figure 4 shows an extended range of simulation results. Both the rheobase and the chronaxie, extracted as parameters from the strength-duration curves, show sensitivity to coupling in this system (Fig. 3c).

To validate the proposed OptoGap approach, we sought a quantitative comparison to the standard GapFRAP method, applied to ChR2-cFB and CMs plated to make lateral connections (Fig. 3d–f). The GapFRAP recovery from photobleaching curves and the extracted time constants^32,33^ are sensitive to the intercellular coupling conditions. The respective diffusion coefficients (D_n_), calculated as the inverse of time constants measured in this study were 0.014, 0.022 and 0.034 for the low, medium and high coupling, respectively. The gapFRAP method was able to resolve coupling strength differences between the low and high levels, but not the middle level. A correlative plot between the mean OptoGap parameters and the GapFRAP-extracted τ shows good correlation between the rheobase of the strength-duration curve and the τ_FRAP_ (r^2^ = 0.9567), while the chronaxie was less correlated with the GapFRAP output (Fig. 3f, Suppl. Figure 5). The chronaxie is more directly linked to the excitability of the cell membrane, including the density of the sodium channels^34^, and this may underlie its lower correlation with the proposed measure of coupling, the light irradiance needed to excite neighboring cells.

Examining the strength-duration curves produced by OptoGap, we note that at short pulses (10 ms), the method is the most sensitive to coupling (see Fig. 3g), and it outperforms GapFRAP in its ability to differentiate between all three coupling conditions. A limitation of our study is that slightly different temperature conditions were used in obtaining data with the two modalities—GapFRAP was performed at room temperature, while OptoGap experiments were done at 30 °C. With a Q10 value of 1.4 for Cx43^35^, it is expected that the gap junctional conductances will change by < 20% between room temperature and 30 °C, which likely will preserve the separation between the different coupling conditions.

## Extending OptoGap to 3D whole heart in silico

**Figure 4:**
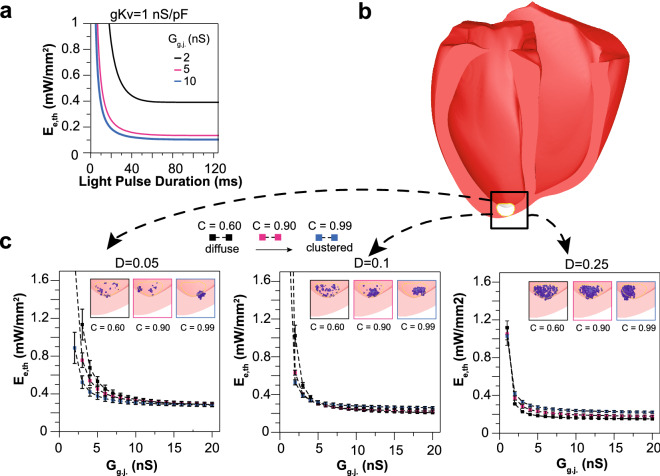
OptoGap applicability to arbitrary cell arrangements in the three-dimensional tissue setting. (**a**) Cellular model of a CM coupled to five ChR2-cFB indicates decreasing E_e,th_ as a function of coupling and pulse duration, as seen in Fig. 3 and as expanded upon in Suppl. Figure 4. (**b**) Schematic of a human heart in which a region at the apex of the left ventricular epicardium (white-coloured region) was infused with ChR2-cFBs coupled to native CMs at different densities (D) and rates of spatial clustering (C). (**c**) Three levels of donor cell density (left to right) were examined. For each value of D, computationally-derived E_e,th_ faithfully reports heterocellular coupling despite very different spatial patterns resulting from variability in C. n = 5 for each combination of D and C parameters, mean ± SE; all individual data points are shown in Suppl. Figure 6.

The experimental validation of OptoGap encouraged computational analysis to further understand the limitations of the method, especially when applied in complex three-dimensional heart settings. Using the cell pair model^23^, where the cFB is optogenetically altered^36,37^, we performed simulations with relative low, medium, and high coupling at G_g.j._ = 2, 5, and 10 nS (resultant V_rest,cFB_ were − 70.9, − 75.6, − 77.3 mV ), applying light of different pulse durations (Fig. 4a). The relationship between E_e,th_ and pulse duration across coupling levels in simulations was similar to the one seen in vitro (Fig. 3a). We then set out to address the important question of whether the OptoGap approach would be applicable in the setting of the three-dimensional heart, where non-myocytes can assume a randomly dispersed pattern. In a geometric model of the human ventricles reconstructed from MRI^38^, we simulated cell delivery of ChR2-nCMs clusters colocalized with native, non-optogenetically modified CMs at the left ventricular apex^29,39^ (Fig. 4b). The clusters covered a range of cell densities (D = 0.05–0.25) and packing arrangements (clustering parameter C = 0.6–0.99). In Fig. 4c, insets of each plot show the schematics of the regions where simulated ChR2-nCM were coupled to CMs; Suppl. Figure 6 presents all simulation data points. OptoGap was simulated by identifying the threshold optical power needed to produce global excitation of the heart through the cell cluster. When the number of cells (i.e. density value) was held constant, spatial distribution did not alter the E_e,th_ readout significantly, as evidenced by the three almost overlapping curves in each plot (Fig. 4c). At higher densities, the relationship between E_e,th_ and G_g.j._ approached a step curve, i.e., the method could detect a critical coupling level of about 2nS, but excitation below that level (around 1nS) was still possible at much higher light levels, similar to the prediction for the optogenetic sensor method. The method is best suited for detecting the integration of sparse arrangement of a few donor cells within the host. These 3D simulations corroborate the applicability of OptoGap and the characteristic relationship between E_e,th_ and gap junctional coupling to a more complex tissue setting. As with other methods discussed above, the sensitivity of OptoGap is best suited to relatively low coupling levels (0–10 nS), which are also of physiological significance for these heterocellular coupling interactions. The readout in the whole heart setting can be optical if regional excitation is of interest, but an electrical readout (an ECG) could also be used) if the contribution of a subset of non-myocytes to the global cardiac excitation is of interest.

## Effects of donor and host cell excitability on OptoGap assay

The detailed example analysis presented in the prior section of ChR2-cFB to CM coupling represents a case where the “donor” (light-sensitized) cells have lower excitability compared to the “host” cells. However, an important conceptual question is if OptoGap could be used with other cell types with different excitability characteristics. There are many potentially relevant scenarios in which this type of analysis could be useful. For example, injection of iPSC-CM in animal hearts following myocardial infarction prompts remuscularization, but can also create a substrate for complex engraftment arrhythmias^7^. Systematic characterization of the time-course of inter-cellular coupling between injected cells and host tissue could help elucidate the underlying mechanisms, which remain poorly understood.

To explore this parameter space, we ran simulations in computational models of tandem cell units designed to probe other configurations, including situations where donor cells are ChR2-expressing human iPSC-CMs instead of cFBs. For this analysis, we defined the OptoGap assay sensitivity metric (σ), which characterizes the log-fold difference in E_e,th_ for different inter-cellular coupling strengths. For example, σ values of + 1 and –1 indicate E_e,th_ values 10 × higher and lower than the threshold under the maximal inter-cellular coupling condition, respectively. See “Methods” section for further detail.

**Figure 5:**
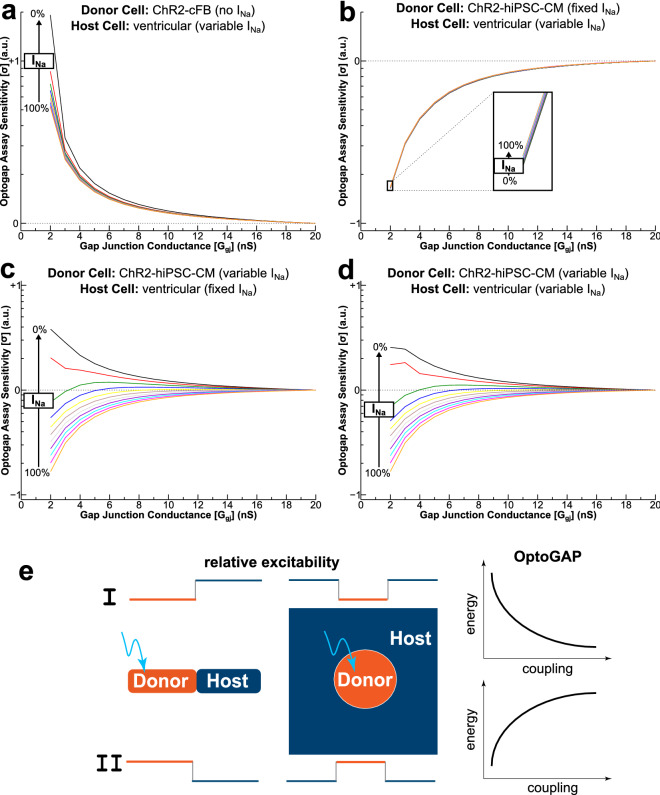
Generalizing OptoGap for different relative excitability between donor (D) and host (H) cells. (**a**) OptoGap assay sensitivity (σ) vs. inter-cellular gap junction conductance for tandem cell unit simulations in which the donor cell has relatively weak electrophysiological excitability (i.e., ChR2-cFB). See text for explanation of σ metric. Different-coloured lines show the same relationship but with gradually reduced host cell excitability (via I_Na_ blockade). (**b**) Same as (a) but for donor cells with a higher baseline level of excitability (ChR2-hiPSC-CM); minor variation between curves. (**c**) Same as (b) but with gradually reduced donor cell excitability instead of host excitability. (**d**) Same as (**b**) but with both donor and host cell excitability reduced by the same amount for each line. (**e**) Conceptual schematics for different D/H configurations. I: When light-sensitive D-cells have lower excitability than the H-tissue (similar to the case of ChR2-cFB among non-transduced cardiomyocytes), the OptoGap test follows an exponentially decreasing curve of energy as function of cell–cell-coupling. Corresponds most closely to the orange line (100% I_Na_) in panel (**a**). II: When light-sensitive D-cells have higher excitability than the H-tissue (similar to the case of cell delivery of ChR2-iPSC-cardiomyocytes into the adult myocardium), the OptoGap test follows an exponentially increasing curve of energy as function of cell–cell-coupling. Corresponds most closely to the orange line (100% I_Na_) in panel (**b**).

For the baseline configuration discussed in the prior section (i.e., ChR2-cFB donor cell; Fig. 5a), we gradually decreased the excitability of the coupled host cell (normal human ventricular myocyte) by simulating progressive blockade of the fast sodium channel (I_Na_). This modification did not alter the shape of the E_e,th_ vs. G_gj_ relationship (i.e., weaker coupling led to higher thresholds for optogenetic stimulation) but markedly improved the assay sensitivity. For example, in the most extreme configuration (0% I_Na_) the ratio between E_e,th_ values for G_gj_ = 2 and 20 nS was 19.207 (2.45 vs. 0.13 mW/mm^2^), resulting in σ = 1.28; for the baseline configuration (100% I_Na_), the analogous σ value was 0.72 (max/min E_e,th_ values of 0.47 and 0.09 mW/mm^2^). Single cell traces of host cell action potentials for this type of TCU model with several excitability/coupling configurations are shown in Suppl. Figure 7a.

Next, we considered scenarios in which the excitability of the donor cells may be higher than that of the relatively inert cFB, e.g., ChR2-iPSC-CMs coupled to native CMs. To do so, we conducted identical tandem cell unit simulations to those discussed in the prior paragraph, but resistively coupled ChR2-cFB donor cells replaced by ChR2-hiPSC-CMs. In all cases involving hiPSC-CM donor cells with unblocked I_Na_, the generalized OptoGap relationship was inverted (i.e., lower G_gj_ values led to weaker rather than stronger E_e,th_ values); this inversion is highlighted most clearly by data shown in Fig. 5b. Moreover, modulation of host cell excitability (via adjustment of I_Na_ level) in the absence of changes in donor cell excitability had a near-indistinguishable effect on assay sensitivity (for G_gj_ = 2 nS, σ ranged from − 0.784 to − 0.777). Representative host cell action potentials for several combinations of G_gj_ and host cell excitability in this configuration can be found in Suppl. Figure 7b.

In contrast, as shown in Fig. 5c, when host excitability was kept fixed and donor cell excitability was reduced (changing I_Na_ from 100 to 0%), the nature and sensitivity of the assay relationship changed dramatically. As ChR2-hiPSC-CM donor cell excitability was reduced, assay behaviour became more similar to the configuration involving (relatively passive) ChR2-cFB donor cells, albeit with reduced assay sensitivity (max. σ = 0.58). Similar results were seen when I_Na_ levels in donor and host cells were adjusted simultaneously and by equal amounts (Fig. 5d). It is important to note that, in some cases, our simulations predict that changing donor cell excitability can degrade assay sensitivity or abolish the monotonic relationship that is essential to proper interpretability of OptoGap. The most striking examples are for cases with 20 and 30% I_Na_ in donor cells (green and blue lines in Fig. 5c,d, respectively), for which σ values are reduced and the E_e,th_ vs. G_gj_ relationship becomes U-shaped, making it impossible to use optogenetic stimulus strength as a measure of intercellular coupling.

Figure 5e shows a conceptual schematic explaining the two broadly defined scenarios of donor-host cell relative excitability and how the assay relationship changes. The computational data shown here suggest that OptoGap has maximal sensitivity to changes in intercellular coupling when light-sensitized donor cells have relatively low excitability and the intrinsic excitability of host tissue is reduced (e.g., via I_Na_ block). The assay can also work properly when donor cells are excitable (e.g., hiPSC-CM), but in this context reduction of host tissue excitability is counter-productive. In all cases, care must be taken to ensure the relationship between E_e,th_ vs. G_gj_ is properly interpreted.

## Discussion and conclusions

We analysed the potential of optogenetic methods for quantitative assessment of electrical heterocellular coupling in the multicellular cardiac setting. None of the existing alternative approaches offers such capabilities. Higher throughput and automation are relatively new aspects to be considered in cardiac electrophysiology^26,37,40^, and only recently optogenetic methods have served as an enabling technology to move in that direction^37,41^.

A scalable quantitative assay of electrical intercellular coupling is of interest for in vitro testing of cell integration or drug effects on coupling. In optimizing stem cell therapy, there has been a strong interest to develop relevant in vitro screening platforms for cell integration, e.g., iPS-progenitor or iPSC-CM integration in engineered cardiac tissues or in human heart slices. Furthermore, there is a concern whether newly developed drugs may inadvertently affect cell–cell coupling and thus be pro-arrhythmic. Conversely, discovery of target small molecules that can restore/augment coupling^42–44^ requires the testing of their effectiveness in a robust and high-throughput manner. For all these cases, a scalable assay^37^, ideally a contactless all-optical quantitative method for coupling such as OptoGap, can enable fast screening that has not been possible before.

For certain heterocellular interactions, e.g., fibroblast-myocyte, in vitro systems may alter the phenotype of the cells and their capacity to couple to each other. The existence and amount of electrical coupling between CM and cFB in vivo and its role in cardiac muscle function has been a controversial topic. In post-infarct injury areas, healthy cFB experience a phenotypic change into myofibroblasts (mFB) in response to excessive mechanical stress in the scar. There has been evidence of higher Cx43 expression in myo-FBs harvested from injury sites or induced by TGF- β1, as well as faster dye transfer with CM than that between normal cFB to CM coupling^4,6,45–47^. This gain in coupling, if electrical, can alter normal wave propagation, be pro-arrhythmic, and generate ectopic activities^3,48,49^. Another example is the sinoatrial node (SAN), where minor alterations in CM-cFB coupling can lead to direct change in SAN pacing rate^50,51^ and are therefore critical for the normal heart rhythm. Other examples from neuroscience exist^17^. Hence, there is a need for appropriate tools such as OptoGap to elucidate the pathologic processes mediated by altered heterocellular electrical coupling in a quantitative way, within the tissue setting. Indeed, recent studies with transgenic mice illustrate implementations of optogenetic probing—by GECIs in donor stem-cell-derived myocytes^2^, by GEVIs in fibroblasts^22^, by ArchT and GECI in heterologous small cell clusters and in the Drosophila brain in vivo^17^, or by ChR2 in macrophages within the intact rodent heart by tracking conduction success or conduction failure^52^. In the area of regenerative medicine, full engraftment of donor cells with the host tissue critically depends on the establishment of proper electrical coupling de novo. Donor cells can be optogenetically transformed prior to delivery and then probed within the myocardium using the technique described and validated here.

To quantify the nature of these heterocellular interactions, measurements need to be done in the three-dimensional cardiac setting. We illustrate that OptoGap may be able to provide a readout of electrical coupling even in these hard-to-control conditions. It provides a direct measure of functional electrical coupling that may depend on factors beyond connexin expression, e.g., cell contact dynamism^46^ and may be influenced by mechanical factors^53^. In general, optogenetic methods are well suited for probing such interactions where cell specificity is needed. Our computational analysis shows that both the use of optogenetic sensors or optogenetic actuators can help in detecting heterocellular coupling. However, there is a difference in the response, such that the action of an optogenetic sensor in non-myocytes yields a predominantly binary outcome—absence or presence of a signal after some very low (< 1 nS) gap junctional coupling is established. In contrast, using light to elicit a response in cardiomyocytes via optogenetically-responsive non-myocytes, coupled to them, provides a more graded readout with the possibility to assess the level of coupling, as validated here.

We demonstrate computationally that the methodology can be extended to cell types with differing excitability, and that the response curve upon optogenetic actuation depends on the donor-host excitability ratio, Fig. 5. When light-sensitive “donor” cells have lower excitability than the “host” tissue, as in the case of ChR2-cFB coupling to non-transduced cardiomyocytes (Fig. 5a,e), the OptoGap test follows an exponentially decreasing curve of energy as function of cell–cell-coupling. Higher-excitability cells can be delivered and probed optically for integration within the native myocardium (Fig. 5b–d), which is particularly relevant to regenerative medicine. In that case, the light-sensitive donor cells have higher excitability than the host tissue and OptoGap test follows an exponentially increasing curve of energy as function of cell–cell-coupling (Fig. 5e). The excitability, captured by the density/conductance of the sodium channels can modulate the actual response (Fig. 5c,d).

There are still several challenges in fully deploying optogenetic methods, including OptoGap, in the whole heart. Light penetration is limited into the dense muscle tissue, especially in the case of blue light^40,54,55^. This problem can be partially resolved by employing red-shifted opsins, such as ReaChR^56^ or CrimsonR^57^, which would permit engaging cells from deeper layers. The short ms-range pulses used here are unlikely to trigger any heating, as analysed earlier^36^, while this may or may not be an issue when pulses are very long, as used in alternative methods^17^. The organ-scale OptoGap experiments presented in this study show that the methodology is capable of differentiating between different degrees of cell–cell coupling between ChR2-nCMs and native CMs arranged in complex 3D patterns, despite the fact that these simulations involved illumination of the endocardial surface only with an accurate model of light attenuation. The second important issue is calibration. OptoGap is most reliable in reporting relative values, i.e., it can detect change in electrical coupling. The absolute optical power values may be used directly, but they may be influenced by a variety of factors other than coupling, therefore proper controls are needed. This limitation (relative reporting) applies to any other method that attempts in vivo quantification so far. Finally, for probing of heterocellular coupling with optogenetic methods in cardiac applications, the challenge is the paucity of selective promoters to target such populations of non-myocytes exclusively as well as the general genetic modification of tissue using viral vectors, as discussed previously^40,55^. The experimental and computational biophysical analysis presented here can help realize such applications, in parallel with the search for better cell-specific genetic targeting within the intact heart.

## Methods

All methods were carried out in accordance with institutional guidelines. Experimental work was done in accordance with the institutional regulations and under an approved protocol by the Institutional Animal Care and Use Committee (IACUC) to obtain primary cardiomyocytes from neonatal rats and an approved protocol by the Institutional Biosafety Committee (IBC) for work with recombinant DNA and viral vectors at Stony Brook University and at George Washington University. No in vivo experiments were performed in this study.

### ChR2 plasmid and virus production

A bacterial stock containing the pcDNA3.1/hChR2(H134R)-eYFP plasmid (developed by the Deisseroth’ laboratory) was obtained from Addgene and amplified in selective Luria–Bertani (LB) medium. Plasmid DNA was extracted and purified using Qiagen HiSpeed Plasmid kit (Qiagen, Valencia, CA), ethanol-precipitated and re-suspended in endotoxin-free water. The purified plasmid was verified by restriction digestion and sequencing and stored at − 20 °C at the obtained concentration (typically 2–4 g/ml). We further incorporated the plasmid into an adenoviral construct (pBR322 backbone) with a ubiquitous CMV promoter^58^. First-generation adenovirus was generated by homologous recombination of the Ad-CMV-ChR2^H134R^-eYFP into pTG3604; further propagation and purification of the virus genomes was done by transfection into HEK293 cells and CsC1 banding.

### Optimization of ChR2 infection protocol

Primary cardiac fibroblasts (cFB) were obtained from the pre-plating steps of ventricular cell isolation (introduced below) and grown to confluence in M199 with 2% FBS supplement in approximately 10 days. They were harvested and transduced with Ad-CMV-ChR2^H134R^-eYFP at optimized dosing^28^. FBs were collected with 0.05% trypsin–EDTA (Gibco Invitrogen, Carlsbad, CA) in Hanks’ balanced salt solution (HBSS, Gibco). After centrifugation, the supernatant was discarded and the cell pellet was re-suspended in culture media with 2% FBS supplement (typically at 1 million cells/ml) and incubated with the virus at multiplicity of infection 2000 (MOI 2000) at 37 °C. Virus containing media was removed promptly as supernatant after 2.5 h of incubation and the cell pellet was re-suspended and maintained in culture media with 10% FBS supplement until co-culture. Expression of ChR2 was validated using eYFP fluorescence as reporter and quantified as (ChR2 positive cells/total nuclei) × 100. Cell mortality was assessed using 2 µM propidium iodide (PI, Invitrogen) stain of dead cells and quantified as PI fluorescence pixels/mm^2^. Both quantification of expression and mortality were processed in an automated image analysis software written with Matlab Image Analysis toolbox.

### Creating ChR2-cFB and CM coupling system and patterning

Prior to co-culture the two cell types, PDMS slabs (Sylgard 184, elastomer to curing agent ratio is 10:1) were cut into 1 cm by 1 cm squares with a circular well of 4 mm diameter in the middle. Glass bottom dishes (In Vitro Scientific, Sunnyvale, CA) were coated with 50 µg/mL human fibronectin (BD Biosciences, San Jose, CA). The PDMS stencil was put in the middle of the glass, and fibronectin was removed prior to seeding cells.

The infected fibroblast population (usually collected after 7–14 days) collected in the same trypsin method introduced earlier, were counted and seeded at density 250k/cm^2^ into the circular well of the PDMS stencil on the glass bottom dish^28^. The cells were put in incubator at 37 °C for 1 h to allow attachment. Then the cell culture media was aspirated away and the stencil was removed without scrapping the cell layer. Immediately, freshly isolated myocytes (procedure done in parallel, introduced below) were plated at density 3.5 × 10^5^/cm^2^ on top and enclosing the focal fibroblast core. Under this patterning regime, the ratio between total fibroblasts (in the focal island) to total myocytes was 1:16.

### Cardiomyocytes culturing

Primary cardiomyocytes (CM) and fibroblasts (cFB) were isolated following the same procedure reported previously^25,31^. Fresh ventricular tissues were harvested from 3–4 day old Sprague–Dawley rats, and digested with 1 mg/ml trypsin (US Biochemicals, Cleveland, OH) in HBSS at 4 °C for 14 h. The pre-treated tissue was further isolated through a 4-repetition serial digestion using 1 mg/ml collagenase (Worthington Biomedical, Lakewood, NJ) in HBSS. After centrifugation, the supernatant containing enzymes was discarded and the cell pellets were re-suspended in culture medium M199 (GIBCO) consisting 12 µM l-glutamine (GIBCO), 0.05 µg/ml penicillin–streptomycin (Mediatech Cellgro, Kansas City, MO), 0.2 µg/ml vitamin B12 (Sigma, St. Louis, MO), 10 mM HEPES (GIBCO), and 3.5 mg/ml d-(+)-glucose (Sigma) supplemented with 10% foetal bovine serum (GIBCO). CM were separated from cFB by a-two-step pre-plating for 45 min each. The purified CM were further counted and plated on fibronectin-treated glass bottom dishes (CM only) or on top of the ChR2-cFB island (co-culture) at 0.35 × 10^6^ cells/cm^2^ (Day 0), and incubated in a humidified environment with 5% CO_2_ at 37 °C. On Day 1, all samples were washed with PBS for 5 min with gentle shaking and continued to be incubated in 10% FBS supplemented media until Day 3, when the culture medium was switched to 2% FBS supplement and exchanged every other day.

### Human iPSC-CPC and iPSC-CM co-culture

Both human iPSC (hiPSC) derived cell types were purchased from Cellular Dynamics International (CDI). Storage and thawing of the cells were done per the company’s guideline. The ratio of hiPSC-CPC to hiPSC-CM was 1:5 for functional traces. hiPSC-CPC were thawed and plated first (day 0). After 24 h, infection was started with dosing the same Ad-CMV-ChR2-eYFP virus (day 1) at MOI 2400 for 5 h incubation. Virus containing media was removed and exchanged with fresh media. After another 24 h, hiPSC-CM were thawed and plated on top of the hiPSC-CPC at the designated cell ratio (day 2). Co-culture media was switched to the hiPSC-CM maintenance media (CDI proprietary) for four days, before functional examination (day 6).

### Immunostaining, imaging, and structural image analysis

All primary cell samples were fixed in 3.7% formaldehyde on Day 4 or Day 5 prior to antibody labelling and imaging. Human iPSC cells were fixed immediately after functional examination on day 6. Cardiomyocytes and iPSC-CM were labelled with mouse anti-alpha-actinin primary antibody (Sigma Aldrich, St. Louis, MO), and Alexa Fluor 647 goat anti-mouse IgG secondary antibody (Invitrogen). hiPSC-CM and hiPSC-CPC nuclei were labelled with rabbit anti-Nkx 2.5 primary antibody (Santa Cruz Biotechnology, Dallas, Texas), and Alexa Fluor 405 goat anti-rabbit secondary antibody (Invitrogen). Connexin43 were labelled with rabbit anti-Cx43 primary antibody (Chemicon, Temecula, CA) and goat anti-rabbit IgG Alexa Fluor 405 secondary antibody (Invitrogen). All antibodies were diluted in 1% bovine serum albumin (Amersham PLC, Amersham, UK).

Imaging was done on Olympus FluoView FV1000 confocal system. To capture the Cx43 between two optical planes, Z-stack images were taken with focal planes 0.5 um apart and sampling speed at 12.5 us/pixel. Panoramic imaging was done in multi-area time lapse software module with sampling speed at 10 us/pixel and 10% overlap.

### Confirmation of optical pacing of iPSC-CM through iPSC-CPC and CM through ChR2-cFB

Action potentials of CM and hiPSC-CM were recorded using the red-shifted voltage-sensitive dye di-4-ANBDQBS (from Dr. Leslie M. Loew, U. Conn). Samples were stained with 35 μM di-4-ANBDQBS for 6 min, followed by a wash of Tyrode’s solution incubation of 6 min. Experiments were done in Tyrode’s solution at room temperature. Imaging was done using a custom-developed attachment to an inverted fluorescence microscope TE2000 (Nikon, Melville, NY) allowing for simultaneous optogenetic stimulation and sensing^37^. Activation light for ChR2 was from a blue LED (Thorlabs, Newton, New Jersey) with a band-pass filter of 470/28 nm (SemRock, Rochester, New York). Excitation light for di-4-ANBDQBS was from a red LED (Thorlabs, Newton, New Jersey) with a band-pass filter 655/40 nm. The blue and red light were combined and directed to fill the back aperture of a 20X objective lens (Nikon, Melville, NY). A 700 nm long-pass emission filter was used to filter the fluorescence signals in front of an EMCCD camera iXon Ultra 897, at 392 fps (Andor, Windsor, CT) over selected regions of interest. Optical pacing of hiPSC-CPC and hiPSC-CM co-culture used 20 ms pulses of 0.03 mW/mm^2^ delivered at 1 Hz. Optical pacing of ChR2-cFB and CM coupling system was delivered via the LED driver using 10 ms pulses of 0.31 mW/mm^2^ at 1 Hz; electrical stimulation of 5 ms, 10 V pulses was delivered via a bipolar point electrode at 1 Hz. Voltage traces were recorded and processed as reported, based on a 2nd degree Savitzky Golay filter after background removal and baseline correction.

### Coupling strength perturbation

Coupling strength was enhanced and reduced by adding gap junction agonist and antagonist, respectively. High coupling condition was created with 1 mM sodium 4-phenylbutyrate (4 PB, Calbiochem, San Diego, CA), diluted in culture media, and put into samples > 48 h prior to experiment time. Low coupling condition was created by sample incubation in 0.5 mM 2-Heptanol (Sigma Aldrich, St. Louis) in Tyrode’s solution (pH 7.4 at 30 °C) for 10 min, followed by continuous perfusion of Heptanol supplement Tyrode’s solution during experiment. Heptanol concentration of 0.5 mM was selected to be less than half the EC50 for cardiomyocytes (about 1 mM)^31^ to avoid non-specific effects.

### GapFRAP assay of coupling

GapFRAP coupling measurements in ChR2-cFB and CM co-culture samples (dispersed cells, not patterned) were done similarly to previously reported procedure^31^ using the Olympus FluoView FV1000. ChR2-cFB and CM coupling system were stained with a low molecular weight dye, calcein-AM (Invitrogen, Carlsbad, CA), at 0.5 μM in Tyrode’s solution for 20 min at room temperature. During image acquisition, both cell types were identified in the phase contrast channel; CM cells had striated appearance and only those that were beating were used. cFB were identified by their thin and flat appearance. Fields of view with a single CM surrounded by cFB only and no other myocytes were selected and scanned at 1% laser power. The single myocyte was then photobleached at 100% laser power for 5–9 s, and images of whole field of view was acquired at every 5 s for 195 s for recovery of fluorescence. During image analysis, auto-detection of the bleached area in the first frame post bleaching was based on sharp contrast edge. Fluorescence intensity of this bleached region on the subsequent frames were extracted as the average of all pixels, and normalized to the first frame post bleaching as zero, $\frac{{F(t) - F(t_{0} )}}{{F(t_{0} )}} \times 100$. Normalized fluorescence recovery data with respect to time were fitted with a perturbation-relaxation equation $F(t) = F_{0} + (F_{\infty } - F_{0} )(1 - e^{ - t/\tau } )$ to extract the time constant, *τ* in Matlab fitting tool box.

### Macroscopic optical pacing and mapping of patterned ChR2-cFB and myocyte coupling system

All samples were paced at 0.5 Hz with Tyrode’s perfusion maintaining 30 °C. Electrical stimulation (10 V, 0.005 s, bipolar) driven by a pulse generator (Ionoptix, Mayopacer) was delivered through platinum electrodes placed at the edge of the petri dish to allow wave propagation throughout the substrate surface. Optical stimulation (2.5 V, TTL pulse) was delivered from below the dish as a collimated beam generated by a fibreoptics-coupled DPSS laser (470 nm, Shanghai Laser) driven by the same pulse generator.

Excitation waves were tracked by fluorescence of calcium-sensitive probe, Quest Rhod-4 (ATT Bioquest, Sunnyvale, CA) through excitation filter 525/40 nm and emission filter 610/75 nm, and registered by an intensified CMOS camera (pco, Germany) at 200 frames/s through high-NA optics. The excitation light for Rhod-4 was delivered as a light sheet parallel to the sample bottom^25^. While watching the camera view, the pulse generator was set at specific pulse duration, and laser intensity was slowly increased until CM fires action potentials. Optical excitation threshold (E_e,th_) was determined as the minimum energy that can sustain CM excitation 1:1 for ten beats. Optical energy (mW/mm^2^) was measured using a digital power meter (Thorlab, Newton, New Jersey)**.**

### Functional data analysis

Using measured strength-duration curves, rheobase and chronaxie were extracted from fits to a parallel RC circuit equation $Irradiance = \frac{Rheobase}{{1 - e^{{ - \frac{t}{Chronaxie}}} }}$ using the curve fitting toolbox in Matlab. Rheobase and chronaxie values of different coupling levels was quantified separately. Each nonlinear least squares fitting had a maximum iteration of 4 × 10^6^ and maximum function evaluations of 6 × 10^6^ with a tolerance of 1 × 10^–12^ for both the fitted model and dependent variable pulse duration. Minimum and maximum differential change was set to 1 × 10^–8^ and 1 × 10^–1^.

Functional signal and image processing were done in custom-developed software (Matlab) for both microscopic and macroscopic recordings. Macroscopic video images of propagation were recorded using the CamWare software (pco, Germany). Image processing and construction of activation maps was done as described previously, using customized software written in Matlab based removal of baseline, second degree filter Savitsky–Golay with kernel size 11^25^. Conduction velocities for ChR2-cFB and CM co-culture samples were measured in both CM_annulus_ and across CM_core_, as the activation waves distance travelled over time.

### Statistics

Statistical analysis was done using GraphPad Prism 9.0.1. For the key irradiance data in Fig. 3a,g, Kolmogorov–Smirnov test confirmed normality of the distributions and parametric tests (ANOVA) with post-hoc Tukey–Kramer correction showed differences between the groups at significance level p < 0.05. For all other cases, groups of three variables were compared using the non-parametric Kruskal–Wallis test with individual differences assessed via Dunn’s multiple comparisons test; see also figure captions.

### Cell- and organ-scale computational simulations

We used our previously validated cardiac optogenetics computational modelling framework to conduct cell- and organ-scale simulations. At the cell scale, we modelled human ventricular myocytes coupled to mammalian ventricular fibroblasts, as described by MacCannell et al.^23^. Briefly, a number of cFB cells (*n*_*FB*_) was electrically coupled to each simulated CM via a lumped representation of gap junctional conductance in parallel *g*_*GJ*_, which ranged in value from 2–20 nS. As in previous studies^23,59^, individual cFB cells were not electrically coupled to each other and all *g*_*GJ*_ values in a particular simulation were identical. All cFB model parameters were implemented as originally published except for (1) characteristic cFB capacitance, which was adjusted to 30.8 pF based on measurements taken from cells used in this study and (2) the conductance of the time- and voltage-dependent cFB K^+^ current (*g*_*Kv*_), which was halved or doubled from its default value (0.25 nS/pF) in some simulations to explore effects of different cFB resting membrane potentials (see Suppl. Figure 4).

Optogenetic transformation of cFB was modelled by adding a biophysically detailed ChR2 photocycle model in parallel to other cFB ionic currents. The expression for light-sensitive current in each cFB was:

$${\text{I}}_{{{\text{ChR2}}}} = {\text{g}}_{{{\text{ChR2}}}} ({\text{O}}_{1} + \gamma {\text{O}}_{2} )G({\text{V}}_{{\text{m}}} ),$$

where *g*_*ChR2*_ was the maximal channel conductance (0.4 nS/pF), *γ* = 0.1 was the ratio between dark- and light-adapted open channel conductance, and *G*(*V*_*m*_) = 10.64–14.64*exp(– *V*_*m*_/42.77) was a voltage-dependent rectification function, as described in our earlier work. Light- and voltage-dependent rates of change for state variables tracking the fraction of channels in open (O1/O2) and closed (C1/C2) states can be found in earlier studies^36,39,60^.

To identify the optogenetic excitation threshold (*E*_*e,th*_) in models of CM/ChR2-cFB tandem cell units (TCU) (*n*_*FB*_ = 5), we first attained a quiescent steady state by simulating 250 s of electrical coupling between the two cell types with no stimuli. Then, as described previously, we used a bisection approach to identify the weakest optical stimulus that elicited an action potential. This process was repeated with different cFB parameters and optical pulse durations (*t*_*stim*_) needed to generate strength-duration curves (Fig. 4a) and other relationships presented in this study.

TCU simulations were also used to assess effects of host and donor cell excitability on the nature and sensitivity of the OptoGap assay (Fig. 5a–d). For simulations involving electrically excitable ChR2-hiPSC-CM donor cells, we used the ventricular variant of the Paci et al. model^61^, as in previously-published multiscale modelling work from our group^62^.

For organ-scale simulations, we used a model of the human ventricles reconstructed from magnetic resonance imaging data^38^. Excitation propagation was simulated via the monodomain formulation^63^. As in our previous study, a hemispherical delivery target (1 cm diameter) was defined near the left ventricular apex and a stochastic delivery algorithm was used to create distributions of hybrid CM/ChR2-cFB TCU (as described above) with different spatial patterns within the region. For each combination of the parameters for ChR2-cFB density, defined as the proportion of the target region not the whole ventricular volume (*D* = 0.05, 0.1, or 0.25), and clustering (*C* = 0.6, 0.9, or 0.99), we created multiple unique distributions (*n* = 5) to ensure that results were not biased by one particular result of the stochastic approach. A ChR2-cFB to CM ratio of *n*_*FB*_ = 20 was used in these simulations because no optogenetic excitation was seen at all for *n*_*FB*_ = 5 due to exaggerated source-to-sink mismatch at the organ scale. Uniform illumination was applied from the endocardial surface local to the delivery site and the exponential decay method (δ = 1.84 mm for blue light with λ = 488 nm) was used to model light attenuation due to energy absorption and photon scattering.

To identify the optogenetic excitation threshold (*E*_*e,th*_) in ventricular models with different spatial distributions of CM/ChR2-cFB TCU we began by applying a sequence of 10 electrical stimuli (72 pA/pF transmembrane current, 2 ms pulse duration, 1 Hz) from the epicardial apex to attain a paced quasi-steady state. Then, *E*_*e,th*_ for a uniform endocardial optical stimulus was identified using the same method described for cell-scale simulations.

For simulations investigating the effects of donor and host cell excitability, we defined the OptoGap assay sensitivity metric [σ]:

$$\sigma = \log_{10} \left[ {\frac{{E_{e,th} (G_{gj} )}}{{E_{e,th} (G_{gj,\max } )}}} \right]$$

where G_gj,max_ was, by definition, 20 nS. We chose to use 20 nS as the maximal G_gj_ value since we found empirically that for all configurations tested the different in E_e,th_ between 19 and 20 nS was negligible (< 1%), suggesting that the relationship between minimal optogenetic stimulus and inter-cellular coupling strength has similar properties to typical strength-duration curves (i.e., E_e,th_ at 20 nS is analogous to the rheobase). The generalized interpretation of this metric is that values farther from 0 in either the positive or negative directions indicate higher assay sensitivity.

All simulations were conducted using the CARP software package^63,64^. A version of this software that is free for academic use is available online (https://opencarp.org). This computational framework has been validated against experimental measurements in numerous previous studies^29,65–72^. Cell-scale simulations were executed on a desktop computer with 8 Intel Core i7 CPUs (3.4 GHz). Organ-scale simulations were executed on 24 Intel Haswell CPUs (2.5 GHz) on the parallel computing resource at the Maryland Advanced Research Computing Center (MARCC).

## Supplementary Information

Supplementary Information.

## Data Availability

All data generated or analysed during this study are included in this published article. Furthermore, we have organized the key experimental data and computational data, and these are made available for download from figshare, https://figshare.com/s/405f6a3f5e8aba4e3f66 (10.6084/m9.figshare.14044976).

## Code availability

Readouts from the experimental preparations described (i.e., threshold light intensities, pulse durations, etc.) are the key measurement of the method itself. No custom code is needed to carry out OptoGap analysis of experimental data. The computational modeling and described can be carried out using software packages that are publicly available for non-commercial use, such as openCARP (https://opencarp.org/). We have made the ChR2 model source code available in our original publication by Williams et al.^36^; it has also been deposited as part of ModelDB (https://senselab.med.yale.edu/modeldb/). Clarification on protocols for computational experiments, including finite element models, electrophysiological model parameters, and model source code will be provided for non-commercial reuse upon all reasonable requests to the authors.
