# Supplementary material for: OptoGap is an optogenetics-enabled assay for quantification of cell–cell coupling in multicellular cardiac tissue
Source: Sci Rep. 2021 Apr 29;11:9310. doi: 10.1038/s41598-021-88573-1 (PMC8085001; doi:10.1038/s41598-021-88573-1)
Supplement: Supplementary file 1 — Supplementary Information. [file 41598_2021_88573_MOESM1_ESM.docx]

**Supplementary Information**

**OptoGap is an optogenetics-enabled assay for quantification of cell-cell coupling in multicellular cardiac tissue**

Patrick M. Boyle^1-5#^, Jinzhu Yu^7#^, Aleksandra Klimas^7,8^, John C. Williams^7^, Natalia A. Trayanova^1,2,6^ and Emilia Entcheva^7,8*^

We modelled the GEVI approach in a cell pair model consisting of 1 cardiac fibroblast^20^ (cFB) and 1 human ventricular cell^65^ (CM) that are linked together at varying gap junction conductance from 0.2 to 20 nS, and explored their respective baseline membrane potentials, potential changes, and action potential durations (APD). As coupling between CM and cFB increased, CM resting potential V_rest,CM_ elevated from -85.05 to -79.27 mV (**Supplementary** **Fig. 1a**), while cFB resting potential V_rest,cFB_ dropped from -52.41 to -78.2 mV (**Supplementary Fig. 1b**). Under electrical stimulation, CM upstroke voltage (change from baseline potential to peak potential) reduced 22% from 133.73 to 104.23 mV (**Supplementary Fig. 1a**), and APD80_CM_ shortened from 387.3 to 165 ms (**Supplementary Fig. 1c**). In the target cell cFB, the upstroke voltage increased by 487% from 15.55 to 91.27 mV (**Supplementary Fig 1b** inset), and duration of 80% repolarization shortened from 351 to 158 ms. These data indicate that V_rest,cFB_ and ΔV_upstroke,cFB_ are two variables sensitive to change in coupling with CM, especially within the range of 0-5 nS. However, optical means of voltage interrogation lacks corresponding potential value. Moreover, the upstroke height in optical imaging of potential change, often represented by change in fluorescence over baseline fluorescence(ΔF/F), is rather small when coupling is < 3 nS, and thus prone to be lost in the poor SNR of existing GEVIs, and cannot be filtered out from the linear multiplication methods in those that lack ratiometric mechanism. Supra-threshold action potential-like depolarization in cFB only started to appear when g_g.j._ was above 3 nS. For detection of coupling in different ranges, the most suitable readout parameter needs to be selected.


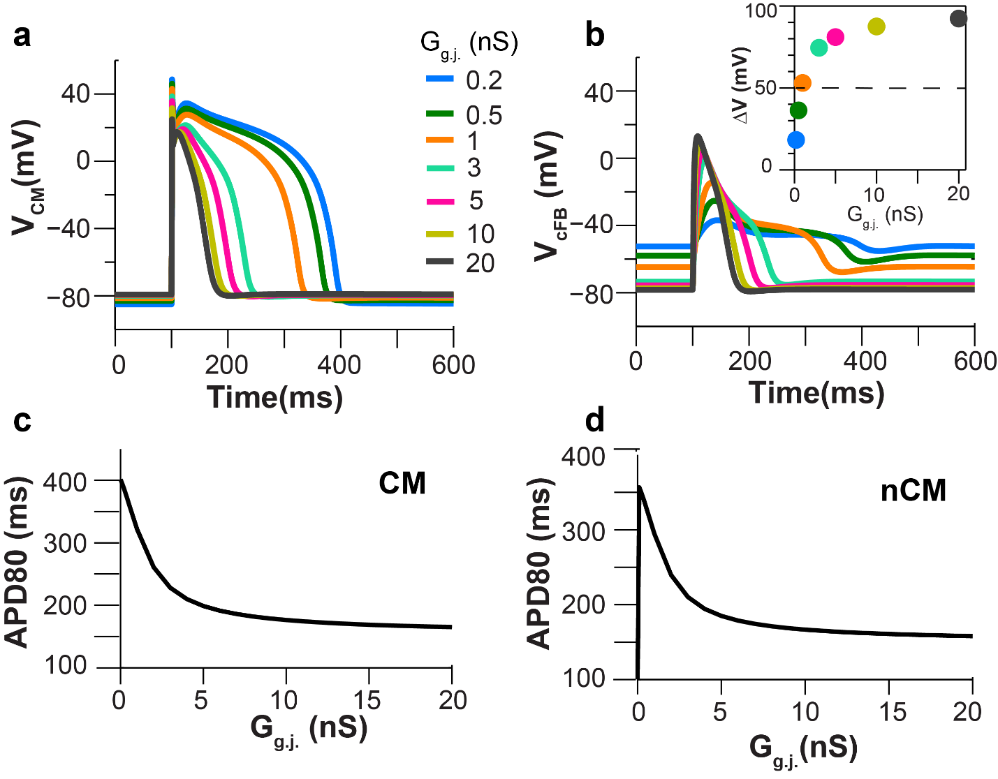


Supplementary Figure 1. **Coupling detected by optogenetics sensor approach**. Membrane potential change in 1 CM (left) coupled to 5 cFBs (right) at selected gap junction conductance (from 0.2-10 nS), in response to a 1 ms, 60 pA/pF current injected into CM. Inset plots maximum potential change in cFBs. **c and d)** Corresponding APD at 80% repolarization of curves in **a** and **b**.


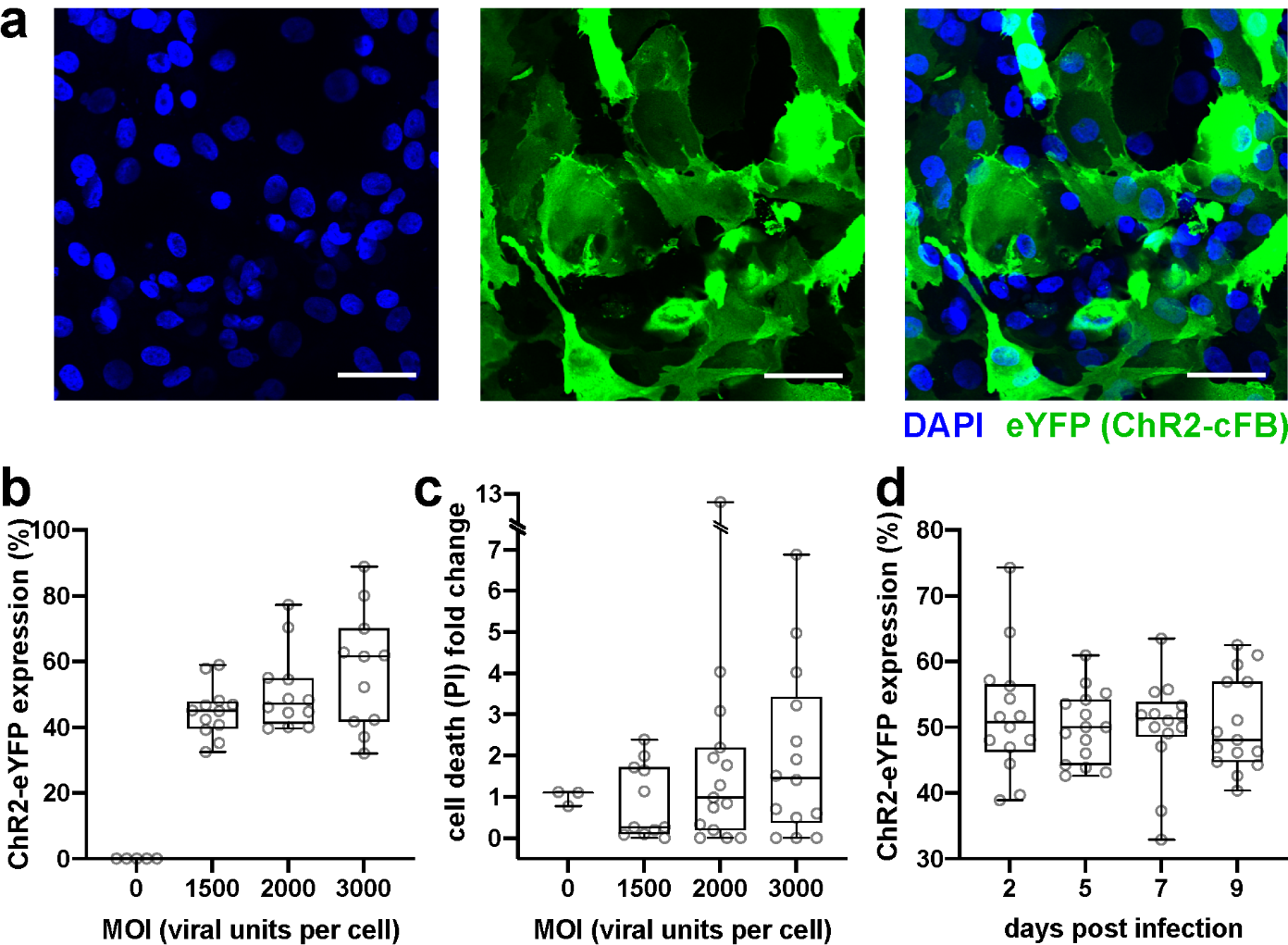


Supplementary Figure 2 **Creating ChR2-cFB a)** Representative images of cFB after infection with Adenovirus-ChR2-eYFP at MOI 2000. Green fluorescence is from the eYFP reporter, blue is from DAPI stain of nuclei. Scale bar is 50 µm. **b)** Expression efficiency improvement by increasing multiplicity of infection (MOI) **c)** The associated cell death quantified by image analysis of infected cells stained with propidium iodide (PI). d) Expression of ChR2-eYFP examined at varies times post infection. Data are from n = 3, 11, 15, 14 fields of view for the different MOIs. All data points are shown, box-whisker data distributions include the median and the interquartile range; the whiskers extend between the minimum and maximum values.

***Regional analysis of conduction velocity over patterned ChR2-cFB and CM coupling system***

Although it may appear counterintuitive, the significant wave acceleration in the CM_annulus_ region can be explained by the eikonal effect, as reviewed in Fast *et al* ^66^.

Over a flat medium that allows straight and non-curved propagation wave fronts, the propagation solely depend on tissue properties, including passive elements such as distribution of gap junctions and cell shapes, and active elements such as membrane conductance and kinetics of ion channels. Without any obstruction, the impulses would travel at steady-state velocity, θ_0_ (**Suppl. Figure 3c,** top left).

However, when the impulse wave front experiences a change in substrate, i.e. encountering the dense ChR2-cFB core, the conduction velocity is altered accordingly depending on the curvature (ρ) and passive properties of the new medium, such as diffusion (D_iff_): $\theta=\theta_{0}+Diff*\rho$ (**Suppl. Figure 3c top right**).

When the wave front meets the lower edge of the ChR2-cFB core, the centre gets slower than the periphery, and a concave wave front curvature is created. The concave wave front improves the net θ due to the positive curvature $\rho=-\frac{1}{r}$ in both CM_annulus_ and CM_core_. However, at the same time, CM_core_ also experiences strong electrotonic load, i.e. low $Diff\propto\frac{1}{R_{i}}$ due to the non-myocytes (high intracellular resistance, R_i_), resulting in opposite effects to the changes brought by curvature. Hence, net $\theta$ changed regionally: paradoxical increase in the CM_annulus_ and unchanged in CM_core_, and explained the trend $\theta_{CM}\left( \theta_{0} \right)\approx\theta_{core} < \theta_{annulus}$.

Adding in a scale of coupling conditions (**Suppl. Figure 3c**, bottom row)

High coupling core: $\theta{=\theta}_{0}(\uparrow)+Diff (\uparrow)\rho$ % change θ_core_ vs θ_annulus_ is 69.2%

Medium coupling core: $\theta{=\theta}_{0}+Diff*\rho$ % change θ_core_ vs θ_annulus_ is 39.6%

Low coupling core: $\theta{=\theta}_{0}(\downarrow)+Diff(\downarrow)\rho$ % change θ_core_ vs θ_annulus_ is 22.1%

Comparison between θ_core_ and θ_annulus_ highlights the difference brought by change in gap junction conductance R_g.j_. since both regions experience the same curvature effect within each coupling conditions.


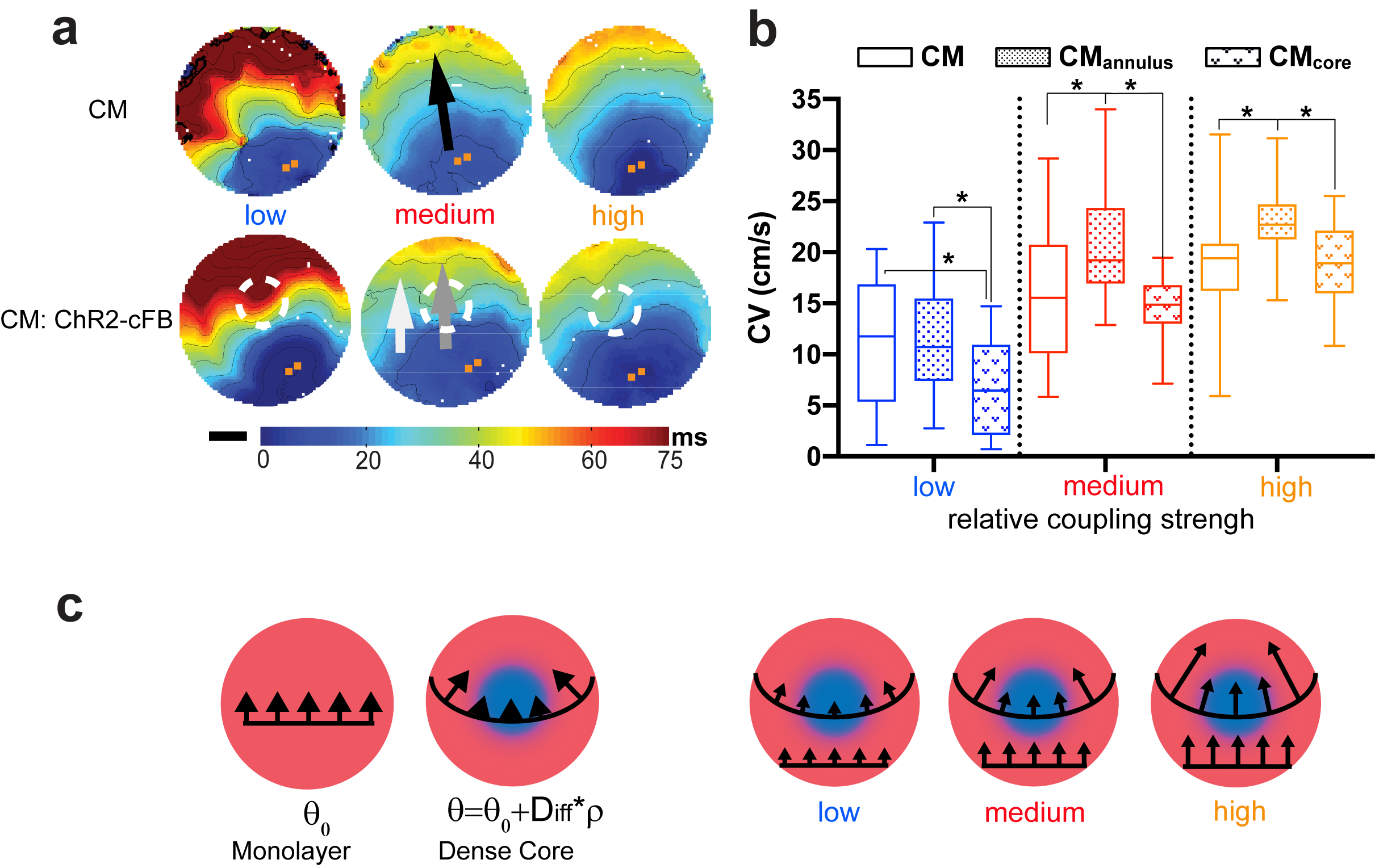


Supplementary Figure 3 **Electrophysiology of the multicellular ChR2-cFB and CM coupling model a)** Activation map of the CM monolayer (top row) and heterogeneous system (bottom row) in response to electrical pacing of 10V in 5 ms pulses, with three relative coupling strengths (low, medium, high), quantified by gapFRAP. Orange square doublets represent the location of electrodes. Arrows indicate the vector of wave front direction, colour of the arrows indicate the region and cell type: black arrow for CM only, grey arrow for CM_core_, and white error for CM_annulus_. Isochrone lines are 10ms apart. **b)** Conduction velocity (CV) derived from the activation maps along the wave front arrows. * indicate significant differences found between same cell type across coupling strength; non-parametric test was used. c) Spatial analysis of CV with respect to geometric characteristics from the presence of a dense core. Top row is a generic comparison between homogenous CM layer and heterogeneous system featuring a dense core. Bottom row represent the heterogeneous system with dense core subject to different coupling strength. Θ_0_ symbolizes the CV of homogeneous CM monolayer, D_iff_ symbolizes the electrical diffusive property of the CM medium, and ρ symbolizes the curvature of the dense core. Black arrows point to the traveling direction of wave front, while their lengths indicate the relative speed. The samples (dishes) used for the different experimental groups were as follows: n = 9 and 14 heptanol treated samples for CM-only and CM:ChR2-cFB; n = 10 and 13 for the control group; and n = 11 and 17 for the 4PB-treated group. The data distributions for CV in (b) are presented as box-whisker plots including the median and the interquartile range; the whiskers extend between the minimum and maximum values.


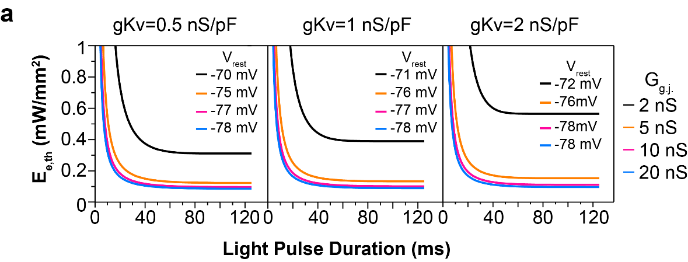


Supplementary Figure 4 **Effect of ChR2-cFBs resting membrane potentials on threshold irradiance E_e,th._** To simulate light pulse excitation experiment in **Figures 3b** and **4a** in wider scope, cell models of 1 CM and 5 ChR2-cFB were run at different ChR2-cFB resting membrane potentials, adjusted by g_Kv_ = 0.5, 1, and 2 nS/pF. While E_eth_ curve was overall shifted up by more negative resting potential, it maintained the exponentially decreasing trend with increasing gap junction conductance.


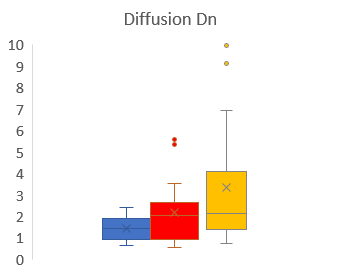


Supplementary Figure 5 **Data distributions shown for the diffusion coefficient (calculated as Dn = 100/τ_FRAP_).** Data used in **Fig. 3f** and **3g** is shown. Blue, red and orange represent the three coupling groups – low, medium and high. Box-whisker plots include the median value and the interquartile range; the whiskers cover 1.5 times the interquartile range, outliers appear as separate points.


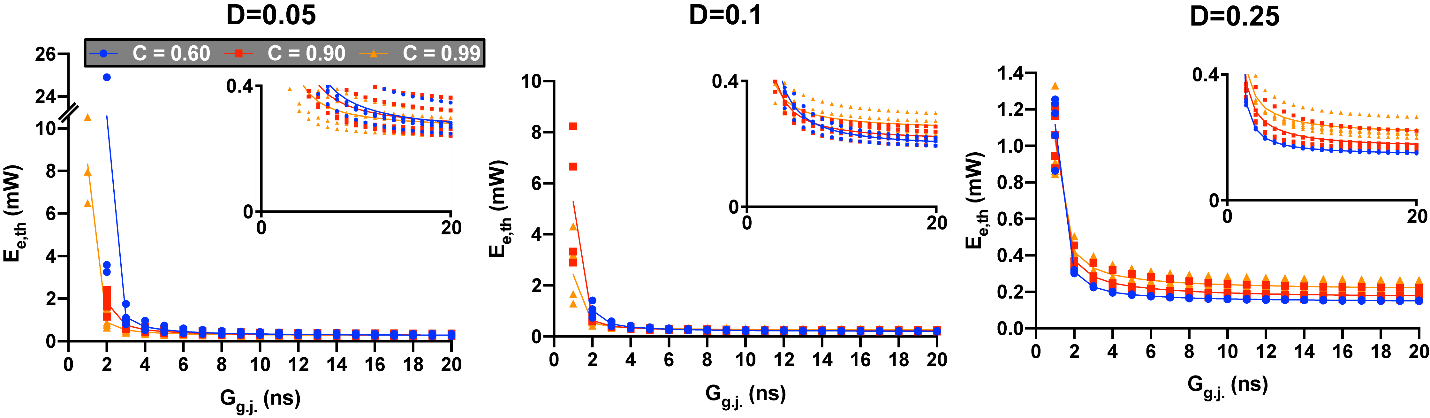


Supplementary Figure 6 **All data points for the five simulations per case (varying density D and clustering C) for the dependence of the excitation threshold, E_e,th_ on the gap junctional coupling, G_g.j_._._** These panels show all simulated data points summarized in the main **Fig. 4c**.


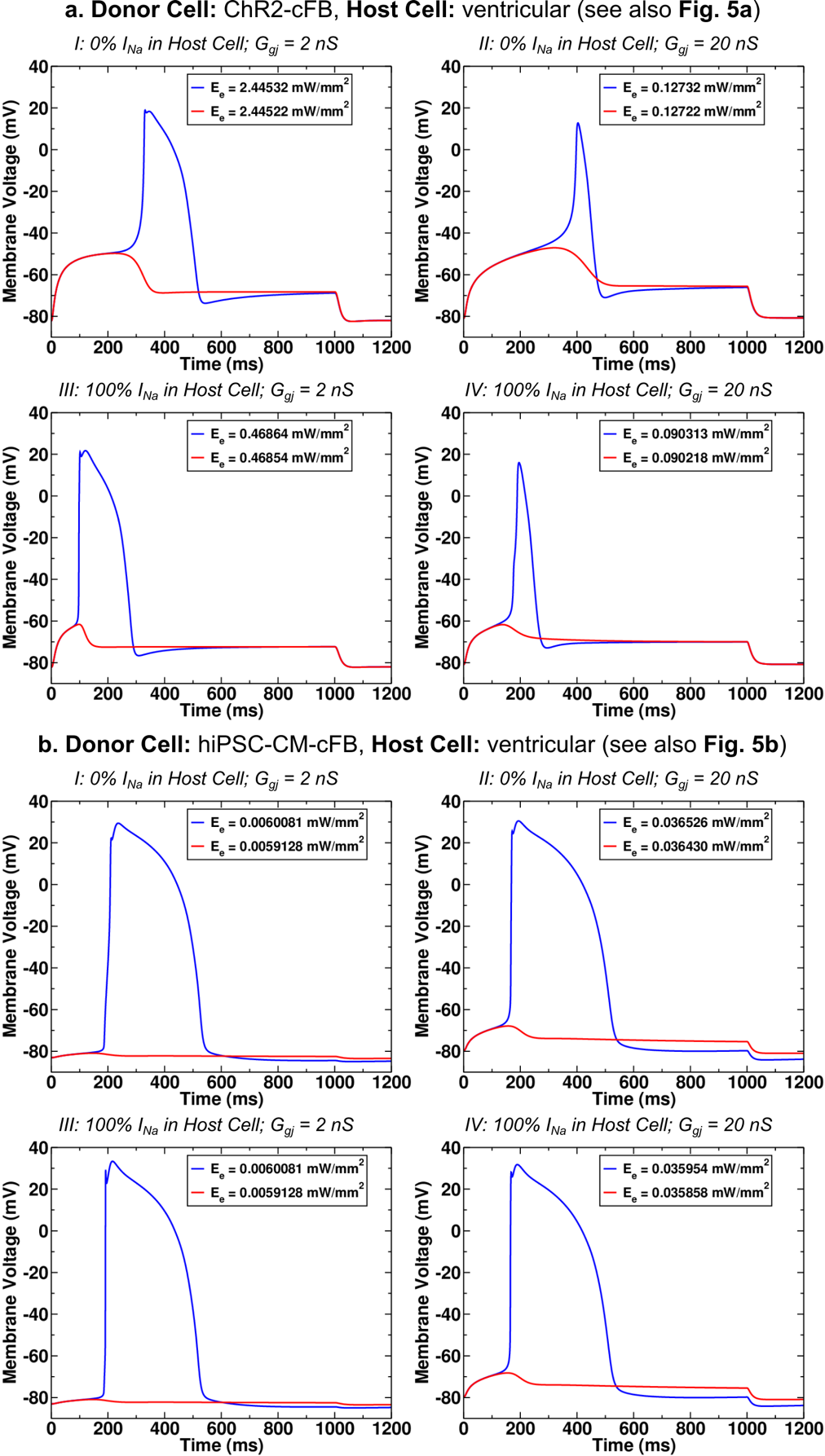


Supplementary Figure 7 **Representative action potential traces from TCU simulations with various combinations of donor/host cell excitability and intercellular coupling.** In each panel, the membrane voltage over time in the host cell is shown. Blue and red traces show the response to optogenetic stimulation for trans-threshold and sub-threshold irradiance (E_e_), respectively. In all cases, illumination onset time is at t = 0 and light pulse duration is 1000 ms.
